# Clinical Accuracy and Length of Percutaneous Pedicle Screw (PPS) Insertion in the Thoracic and Lumbar Spine Under Two-Dimensional Fluoroscopy: A Comparative Study of PPS Placement With Guidewireless and Conventional Guidewire Systems

**DOI:** 10.7759/cureus.92307

**Published:** 2025-09-14

**Authors:** Yuta Doi, Satoshi Baba, Mitsumasa Hayashida, Takao Mae

**Affiliations:** 1 Orthopaedic Surgery, Saga-Ken Medical Centre Koseikan, Saga, JPN; 2 Orthopaedic Surgery/Spine Surgery, Saga-Ken Medical Centre Koseikan, Saga, JPN

**Keywords:** guidewireless system, percutaneous pedicle screw, screw insertion accuracy, screw insertion depth, thoracolumbar spine, two-dimensional fluoroscopy

## Abstract

Introduction

Minimally invasive spine surgery, particularly percutaneous pedicle screw (PPS) placement, has gained popularity. The VIPER PRIME™ Spine System (DePuy Synthes, Raynham, Massachusetts, United States), introduced in 2018, was designed to eliminate guidewire complications. This study compared the accuracy and insertion length of PPS placement using the Viper Prime system and the conventional guidewire system under two-dimensional fluoroscopy.

Methods

A total of 46 patients (30 men, 16 women; mean age 72.9 years) who underwent PPS fixation between 2018 and 2022 were included. Patients were divided into two groups: the VIPER PRIME group (N-group) and the conventional group (C-group). A total of 263 screws were inserted in the N-group and 133 screws in the C-group. All procedures were performed using two-dimensional fluoroscopic guidance.

Results

The N-group demonstrated 263/263 (100%) screws placement accuracy, with all screws classified as Zdichavsky grade Ia or Ib (used with permission from Springer Nature). The C-group achieved 131/133 (98.5%) screws placement accuracy. No severe complications or screw revisions occurred in either group. Analysis of screw length showed that only 59/263 (22%) screws in the N-group exceeded 85% of the vertebral body length, compared to 82/133 (62%) screws in the C-group.

Conclusion

The VIPER PRIME system demonstrated accuracy comparable to the conventional system in PPS placement under two-dimensional fluoroscopy. Its high precision is likely attributable to the ease of positioning and the relatively thick stylet, which is designed to be firmly grasped at a short length, reducing deflection during insertion. However, the integrated screw design may lead to the use of shorter screws to avoid anterior cortical breach. In patients with osteoporosis or those requiring stronger fixation, careful preoperative planning using three-dimensional computed tomography is recommended to ensure optimal screw length and placement.

## Introduction

Minimally invasive spine surgery (MISS) techniques have been increasingly adopted for thoracolumbar spinal disorders, offering reduced soft-tissue disruption while achieving outcomes comparable to open surgery [1]. In particular, percutaneous pedicle screw (PPS) fixation has gained popularity as a thoracolumbar fixation surgery [1]. Compared to traditional open instrumentation, the percutaneous approach is associated with less muscle injury, decreased intraoperative blood loss, lower infection rates, and faster recovery in appropriately selected patients [2-6].

Conventional PPS placement relies on guidewires to direct cannulated pedicle screws into position. However, the use of guidewires introduces additional challenges and risks. Guidewires can bend or break during tapping, potentially prolonging operative time and increasing fluoroscopic exposure. More importantly, an inadvertent advance of the guidewire beyond the vertebral body can cause catastrophic damage to anterior structures. One serious potential complication is great vessel or bowel injury caused by the guidewire’s anterior migration through the cortical wall [7]. Zhao et al. reported a 5.9(%) overall complication rate in a large series of 781 PPS cases, including instances of intraoperative guidewire breakage and even an abdominal arterial injury from anterior guidewire perforation [8]. Meticulous technique is required to avoid such events, prompting interest in safer alternatives to the guidewire method. The VIPER PRIME™ Spine System (DePuy Synthes, Raynham, Massachusetts, United States), introduced in 2018, was designed to eliminate guidewire complications. However, limited studies have compared the clinical pedicle screw accuracy and length of insertion using the guidewireless system versus the conventional guidewire system.

This study evaluated and compared the placement accuracy of the guided wireless PPS system with that of the conventional guidewire system using two-dimensional fluoroscopy.

## Materials and methods

This retrospective study included patients who underwent PPS fixation using two-dimensional fluoroscopy at Saga-Ken Medical Centre Koseikan, Saga, Japan, between April 2018 and December 2022 and had available postoperative computed tomography (CT) images for evaluation. All patient data were anonymized to protect personal information in accordance with applicable privacy regulations and ethical standards.

A total of 46 patients were included, of which 30 patients (N group) underwent screw insertion using the guidewireless system, while 16 (C group) used the conventional guidewire system. A total of 263 and 133 screws were inserted in N- and C-groups, respectively. The clinical accuracy of screw insertion was evaluated according to Zdichavsky’s scoring system [9], which was used with permission from Springer Nature.

Optimal screw positioning was classified as Types 1a and 1b. Positions 2a and 2b should be assessed for stability, but typically do not require revision. Positions 3a and 3b indicate poor placement and may necessitate screw revision depending on stability and possible neurological damage [9]. Screw length was evaluated based on the criteria described by Heintel et al [10]. Screws with a length exceeding 85% of the total vertebral body were rated as good, whereas those exceeding 90% were rated as excellent. Data were collected on screw length, screw placement accuracy, and related parameters from postoperative imaging (CT images). 

Surgical procedure

Surgeries were performed according to the respective device manuals: the VIPER PRIME system for the N group and each manufacturer’s spinal fixation system for the C group. A summary of both procedures is presented in Table 1.

**Table 1:** Surgical procedure steps in the conventional K-wire (C) and guidewireless (N) groups

Step	N Group	C Group
1	Make a skin incision	Make a skin incision
2	Insert screw-loaded inserter to the pedicle entry point	Advance needle or probe to the pedicle
3	Advance stylet into the pedicle	Confirm entry into vertebral body and insert guidewire
4	Advance the screw	Remove needle or probe
5	Remove inserter and stylet	Insert tap over guidewire
6	—	Remove tap
7	—	Insert screw
8	—	Remove guidewire
9	—	Remove driver from the screw

Statistical analysis

All analyses were conducted using R software version 4.2.2 (R Foundation for Statistical Computing, Vienna, Austria, https://www.R-project.org/), and significance was set at p < 0.05. Comparisons between the two groups were performed using Fisher's exact test for nominal variables and the Mann-Whitney U test for continuous variables.

## Results

**Figure 1:**
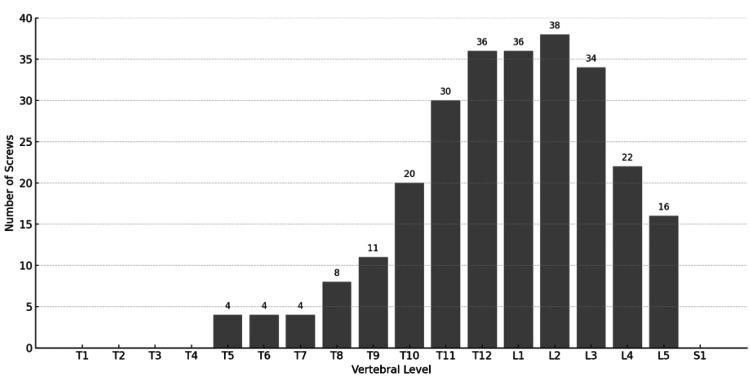
Distribution of percutaneous pedicle screws inserted in the N-group by vertebral level (N = 263, with the highest frequency at L2)

**Figure 2:**
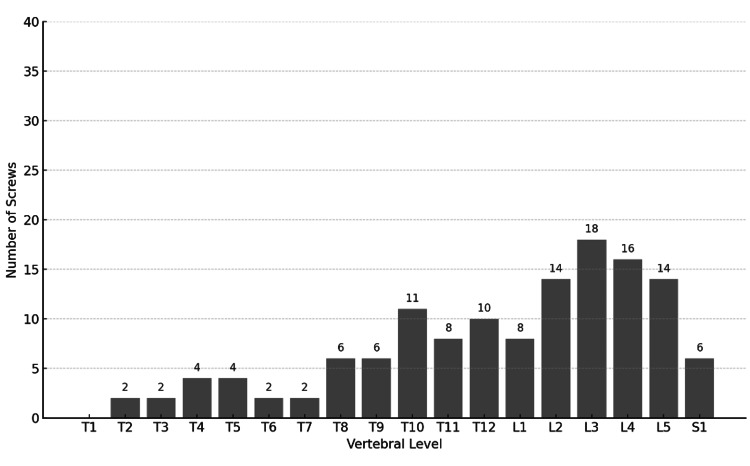
Distribution of percutaneous pedicle screws inserted in the C-group by vertebral level (N = 133, with the highest frequency at L3).

A total of 46 patients (30 men and 16 women, mean age 72.9 years) were included in the study. There were no significant differences between the N Group (guidewireless) and the C Group (conventional guidewire) in terms of age, sex, or PPS insertion levels, as shown in Table 2 and Figures 1, 2.

**Table 2:** Basic data for the guidewireless (N) and the conventional guidewire group (C)

Variable	N-group	C-group
Sex (female/male), n	10/20	6/10
Age (years), median (range)	74.5 (60-81)	70 (62.5-81)
Number of screws per person, median (range)	8 (8-11.5)	8 (6-10.25)

Accuracy

**Figure 3:**
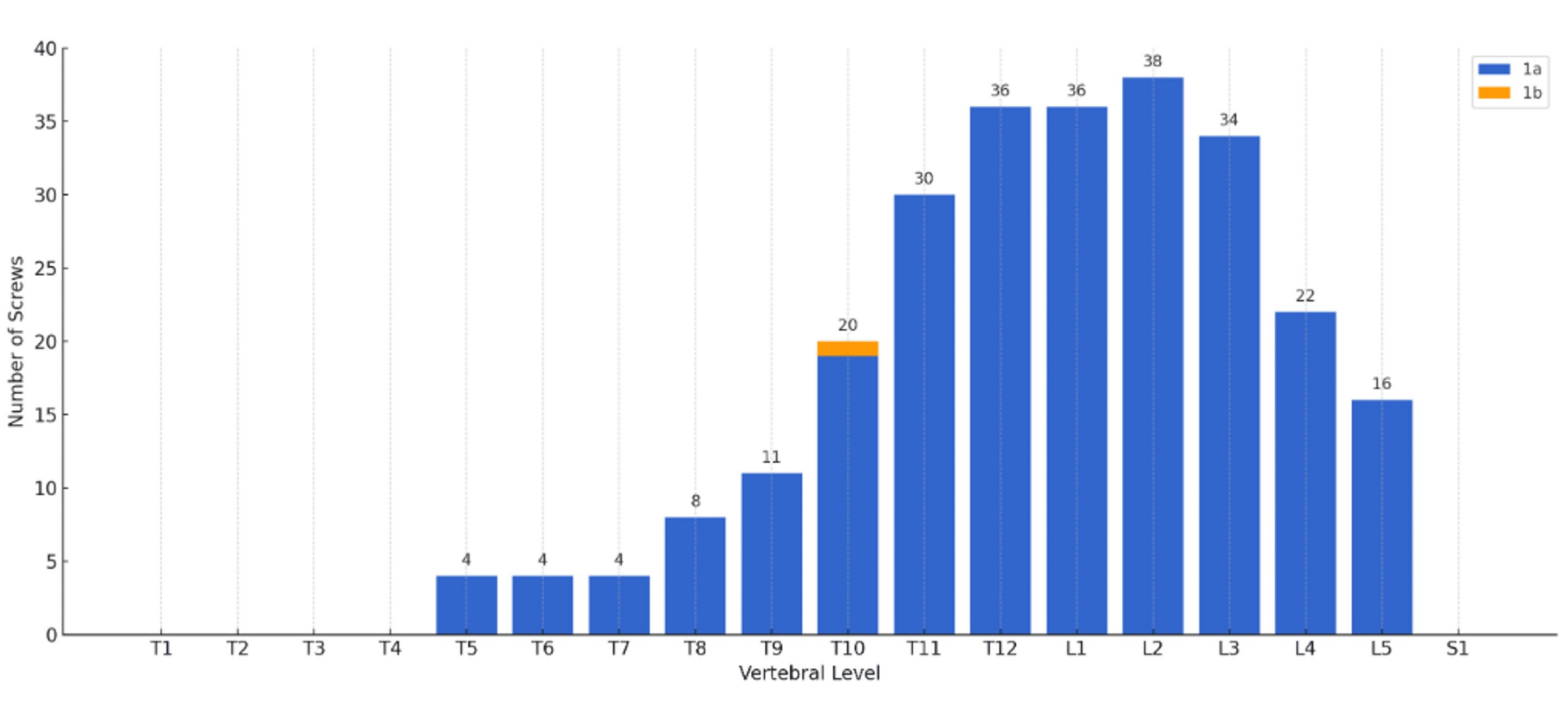
Clinical accuracy of screw insertion assessed by Zdichavsky’s scoring system in the N-group (Grade Ia＝262 (99.6%) screws, Ib=1 (0.4%) screws). Zdichavsky’s scoring system [9] is not part of the governing open access license but has been used with permission from Springer Nature.

**Figure 4:**
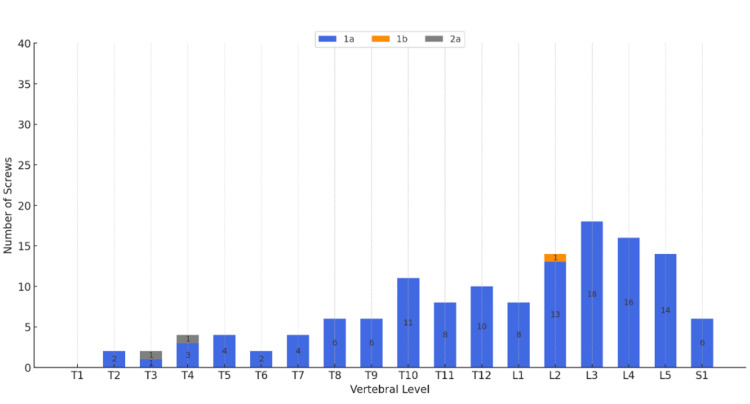
Clinical accuracy of screw insertion assessed by Zdichavsky’s scoring system in the C-group (Grade Ia＝130 (97.7%), Ib= 1 (0.8%), IIa =2 (1.5%)） Zdichavsky’s scoring system [9] is not part of the governing open access license but has been used with permission from Springer Nature.

According to Zdichavsky’s scoring system, a total of 262 (99.6%) screws in the N-group and 130 (97.7%) screws in the C-group were classified as grade 1a, while one screw in each group (0.4% in the N-group, 0.8% in the C-group) was classified as grade 1b (Table 3). All screws in the N-group were placed in clinically optimal positions (grades 1a and 1b) (Figure 3). In the C-group, two screws (1.5%) were classified as grade 2a, indicating suboptimal placement (Figure 4). However, no neurological complications were observed, and no screw revisions were required in either group.

**Table 3:** Clinical accuracy of screw insertion assessed by Zdichavsky’s scoring system (Unit: Screws) Zdichavsky’s scoring system [9] is not part of the governing open access license but has been used with permission from Springer Nature.

Classification	N-group	C-group
1a	262	130
1b	1	1
2a	0	2
2b	0	0
3a	0	0
3b	0	0
Total	263	133

Length

**Figure 5:**
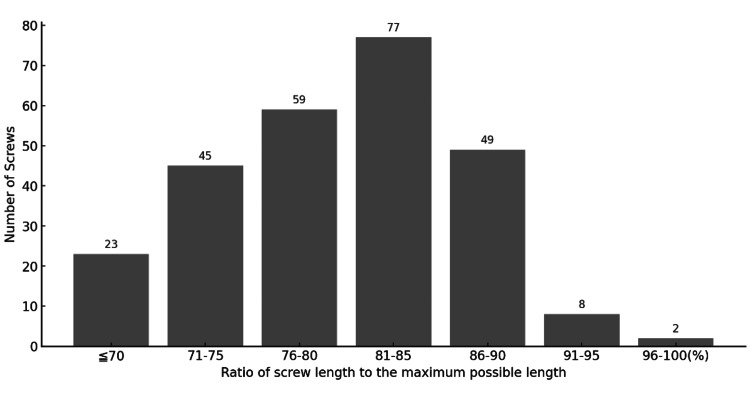
Ratio of screw length to the maximum possible length in the N-group. Screw length was evaluated based on the criteria described by Heintel et al. [10]. Two screws achieved nearly 100% of the maximum possible length, eight screws achieved 91–95%, 49 screws achieved 86–90%, and 77 screws achieved 81–85% of the maximum possible length.

Screws reaching at least 85% of the vertebral body length were classified as “good,” and those reaching 90% or more were considered “excellent” (Table 4). Among the 263 screws in the N-group, two achieved nearly 100% of the possible length, eight reached 91-95%, 49 reached 86-90%, and 77 reached 81-85% (Figure 5). Overall, 59 (22%) screws in the N-group were classified as good or excellent.

**Table 4:** Ratio of screw length to the maximum possible length. Screw length was evaluated based on the criteria described by Heintel et al. [10]

Length (%)	N group, n	C group, n
≦70	23	2
71-75	45	1
76-80	59	13
81-85	77	35
86-90	49	46
91-95	8	19
96-100	2	17

**Figure 6:**
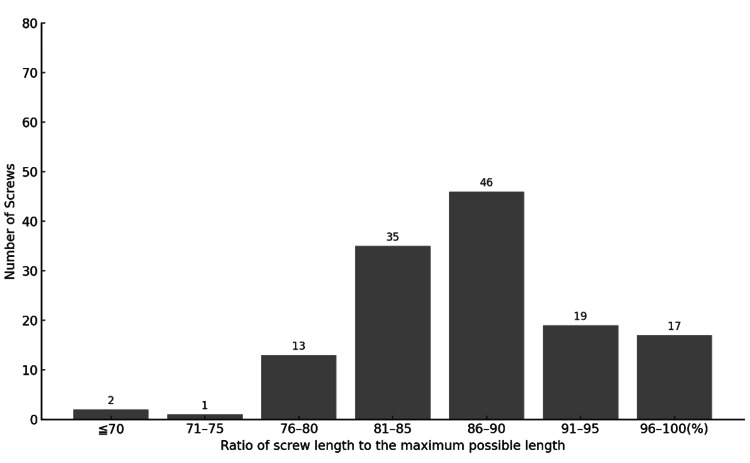
Ratio of screw length to the maximum possible length in the C-group. Screw length was  evaluated based on the criteria described by Heintel et al. [10]. A total of 17 screws achieved nearly 100% of the maximum possible length, 19 achieved between 91–95%, 46 were within 86–90%, and 35 screws were between 81–85% of the maximum possible length.

In the C-group, out of 133 screws, 17 reached nearly 100%, 19 reached 91-95%, 46 reached 86-90%, and 35 reached 81-85% of the maximum length (Figure 6). A total of 82 (62%) screws in the C-group were rated as good or excellent.

## Discussion

In the VIPER PRIME guidewireless group, all pedicle screws were inserted into the optimal positions classified as Zdichavsky's grade 1. In contrast, in the conventional group, 98.5% (130/132) of the screws were placed in positions classified as Zdichavsky's grade 2. No severe complications were observed. Ohmori et al. reported an accuracy rate of 85.7% (150/175 screws) for screw placement in the guidewireless group compared to 75.0% (123/164 screws) in the guidewire group, with a difference of 10.7% between the groups [11]. Compared with previous studies, the clinical accuracy of screw placement using the guidewireless technique was at least comparable to conventional methods. The comparable accuracy observed in the VIPER PRIME group may be attributed to several factors. In addition to the ease of positioning, the relatively thick stylet, designed to be securely grasped at a short length, likely minimizes deflection during insertion. This characteristic may have contributed significantly to the high precision of the screw placement.

However, the screw lengths of the VIPER PRIME group tended to be shorter. Screw length plays a crucial role in the fixation strength of PPS. Biomechanical and clinical studies have shown that longer screws generally provide greater holding power. For instance, a finite element analysis study by Matsukawa et al. demonstrated that increasing the diameter and length of screws significantly improves axial pullout strength and spinal fixation strength, while also reducing stress on the surrounding bone [12]. In osteoporotic models, shorter screws tend to reduce fixation strength and pullout resistance. Clinically, adequate screw insertion depth into the vertebral body is essential. Matsukawa et al. studied posterior fixation in patients with degenerative spondylolisthesis and found that groups with greater screw insertion depth (≥54.2% of the L4 vertebral body length) had significantly higher fusion rates [13]. Receiver operating characteristic (ROC) analysis suggested that an insertion depth of about 55% or more of the vertebral body length was optimal for achieving good fusion outcomes. A previous study on PPS insertion using conventional techniques reported that 85% of screws exceeded 85% of their maximum possible length [3]. Additionally, previous studies on pedicle screw insertion depth have recommended a depth of approximately 80% of the vertebral body length [14].

In the VIPER PRIME group, the integrated screw design likely influenced the selection of shorter screw lengths. Because screw lengths must be determined preoperatively, shorter screws are often selected to prevent anterior protrusion beyond the vertebral body. Anterior vertebral injuries may result in severe complications, including damage to the anterior structures and major abdominal vessels. However, achieving rigid fixation requires the screw to be inserted near the anterior cortical bone of the vertebral body. Hirano et al. demonstrated that screws reaching the anterior cortical bone of the vertebral body can increase the pullout strength by 20-25% [15]. For cases involving osteoporotic bone or those requiring more robust fixation, meticulous preoperative planning using three-dimensional computed tomography (3D-CT) imaging is essential.

3D-CT is useful in preoperative planning for PPS insertion, as it allows for the detailed visualization of the vertebrae and the optimal screw trajectory in three-dimensional space. This advanced imaging technique aids in the precise determination of screw length, diameter, entry point, and trajectory. Ozaki et al. demonstrated that preoperative planning software utilizing 3D-CT allows for the optimization of screw size and trajectory tailored to each patient's unique anatomy [16]. The software provides a virtual simulation of screw placement, offering an opportunity to verify entry points and ensure the trajectory is correctly planned before surgery. Similarly, Park et al. highlighted the use of CT-based preoperative simulation systems for planning screw insertion angles, lengths, and diameters, leading to improved outcomes compared to traditional free-hand methods [17]. Especially in patients with osteoporosis or advanced age, the risk of screw loosening and pullout may be increased due to weaker bone quality. Preoperative 3D-CT planning may allow for the selection of the optimal screw size and trajectory tailored to the specific bone quality, potentially contributing to improved fixation strength.

This study has several limitations. First, it was a retrospective analysis conducted at a single institution, which may limit the generalizability of the findings. Second, the sample size was relatively small, especially in subgroup comparisons, and the number of screws varied between groups, potentially introducing selection bias.

## Conclusions

The VIPER PRIME group demonstrated pedicle screw placement accuracy comparable to that of the conventional guidewire method under two-dimensional fluoroscopy. However, screw length tended to be shorter, which may affect fixation strength. Incorporating preoperative planning with 3D-CT may enable the selection of maximal safe screw lengths, thereby optimizing biomechanical stability while maintaining procedural safety. Clinically, these findings highlight the importance of integrating advanced imaging-based planning when adopting guidewireless systems, particularly in patients with compromised bone quality or those requiring enhanced fixation strength.
